# Heme oxygenase-1-mediated neuroprotection in subarachnoid hemorrhage via intracerebroventricular deferoxamine

**DOI:** 10.1186/s12974-016-0709-1

**Published:** 2016-09-13

**Authors:** Robert H. LeBlanc, Ruiya Chen, Magdy H. Selim, Khalid A. Hanafy

**Affiliations:** 1 Department of Neurology, Beth Israel Deaconess Medical Center, Harvard Medical School, 3 Blackfan Circle, Boston, MA 02140 USA; 2 Division of Neurointensive Care Medicine, Beth Israel Deaconess Medical Center, Harvard Medical School, 3 Blackfan Circle, Boston, MA 02140 USA

**Keywords:** Deferoxamine, Heme oxygenase, Immunology, Intracerebroventricular, Microglia, Subarachnoid hemorrhage, Vasospasm-independent

## Abstract

**Background:**

Subarachnoid hemorrhage (SAH) is a devastating disease that affects over 30,000 Americans per year. Previous animal studies have explored the therapeutic effects of deferoxamine (DFX) via its iron-chelating properties after SAH, but none have assessed the necessity of microglial/macrophage heme oxygenase-1 (HO-1 or Hmox1) in DFX neuroprotection, nor has the efficacy of an intracerebroventricular (ICV) administration route been fully examined. We explored the therapeutic efficacy of systemic and ICV DFX in a SAH mouse model and its effect on microglial/macrophage HO-1.

**Methods:**

Wild-type (WT) mice were split into the following treatment groups: SAH sham + vehicle, SAH + vehicle, SAH + intraperitoneal (IP) DFX, and SAH + ICV DFX. For each experimental group, neuronal damage, cognitive outcome, vasospasm, cerebral and hematogenous myeloid cell populations, cerebral IL-6 concentration, and mitochondrial superoxide anion production were measured. HO-1 co-localization to microglia was measured using confocal images. Trans-wells with WT or HO-1^−/−^ microglia and hippocampal neurons were treated with vehicle, red blood cells (RBCs), or RBCs with DFX; neuronal damage, TNF-α concentration, and microglial HO-1 expression were measured. HO-1 conditional knockouts were used to study myeloid, neuronal, and astrocyte HO-1 involvement in DFX-induced neuroprotection and cognitive recovery.

**Results:**

DFX treatment after SAH decreased cortical damage and improved cognitive outcome after SAH yet had no effect on vasospasm; ICV DFX was most neuroprotective. ICV DFX treatment after SAH decreased cerebral IL-6 concentration and trended towards decreased mitochondrial superoxide anion production. ICV DFX treatment after SAH effected an increase in HO-1 co-localization to microglia. DFX treatment of WT microglia with RBCs in the trans-wells showed decreased neuronal damage; this effect was abolished in HO-1^−/−^ microglia. ICV DFX after SAH decreased neuronal damage and improved cognition in *Hmox1*^*fl/fl*^ control and *Nes*^*Cre*^*:Hmox1*^*fl/fl*^ mice, but not *LyzM*^*Cre*^*:Hmox1*^*fl/fl*^ mice.

**Conclusions:**

DFX neuroprotection is independent of vasospasm. ICV DFX treatment provides superior neuroprotection in a mouse model of SAH. Mechanisms of DFX neuroprotection after SAH may involve microglial/macrophage HO-1 expression. Monitoring patient HO-1 expression during DFX treatment for hemorrhagic stroke may help clinicians identify patients that are more likely to respond to treatment.

**Electronic supplementary material:**

The online version of this article (doi:10.1186/s12974-016-0709-1) contains supplementary material, which is available to authorized users.

## Background

Over 30,000 Americans will fall victim to an aneurysmal subarachnoid hemorrhage (SAH) this year; nearly half of these patients will die within 6 months [1, 2]. Of those SAH survivors, approximately 50 % will develop severe cognitive and functional deficits [3, 4]. Although the majority of research in SAH has focused on the treatment of vasospasm, only nimodipine has been shown to improve outcome [5, 6]. Multiple clinical trials have demonstrated that even when vasospasm was effectively treated, morbidity and mortality were not ameliorated [7–9]. These studies suggest that neurological injury can be vasospasm-independent. Further research aimed at mitigating this heme-induced cerebral inflammatory response is required [10].

In SAH, the heme from blood spilled into the subarachnoid space is metabolized by heme oxygenase (HO), generating excess free iron [11]. This iron is hypothesized to enable cell membrane damage via free radicals [12] with studies showing a causal relationship between unbound iron and brain injury following SAH [13]. Deferoxamine (DFX), an iron-chelating agent, has been shown to be neuroprotective in various hemorrhagic models via several mechanisms [12, 14]. Previous studies using a rat model of SAH showed decreased brain edema, oxidative stress, and neuronal apoptosis after DFX treatment [15, 16]. However, none of these studies assess the necessity of heme oxygenase-1 (HO-1 or Hmox1) in DFX neuroprotection nor has the efficacy of intracerebroventricular administration been fully examined. Our lab previously showed microglia to be critical in red blood cell-induced neuroinflammation [10], and most recently, we found microglial HO-1 to be neuroprotective after SAH in a mouse model [17]. We hypothesized that microglial/macrophage HO-1 is critical for DFX neuroprotection and that intracerebroventricular administration would provide superior neuroprotection in a mouse model of SAH.

We undertook the current set of experiments to first compare the effects of systemic versus intracerebroventricular injection of DFX on neuronal damage, vasospasm, pro-inflammatory and oxidative biomarkers, and immune cell populations in a mouse model of SAH; second, to see if microglial/macrophage HO-1 is sufficient for DFX neuroprotection; and third, to compare the effects of cell-specific HO-1 knockouts on DFX neuroprotection and cognitive outcome. Our study provides a platform for the potential translation of DFX treatment into the SAH patient population.

## Methods

### Animal information and anesthesia

All experimental procedures were approved by the Institutional Animal Care and Use Committee (IACUC) of Beth Israel Deaconess Medical Center (BIDMC). The facility is accredited by the Association for Assessment and Accreditation of Lab Animal Care and fully complied with all Federal, State and Local Law. Animals were housed at BIDMC and fed a standard rodent diet ad libitum with 24-h access to either water and/or hydrogel while kept on a 12-h light/12-h dark cycle. All surgical manipulations were performed under general anesthesia with ketamine (10 mg/kg) and xylazine (4 mg/kg), and buprenorphine (50 μg/kg) was systemically administered. All mice used were male on a C57BL/6 background (The Jackson Laboratory). Cell-specific HO-1 knockout in myeloid cells (*LyzM*^*Cre*^:*Hmox1*^*fl/fl*^) and astrocytes and neurons (*Nes*^*Cre*^:*Hmox1*^*fl/fl*^) was achieved as previously described by our lab [17]. All mice had similar fur color and were approximately the same size and weight. The average mouse weight was 25 g (0.025 kg) with a weight range of 24 g (0.024 kg) to 27 g (0.027 kg). Wild-type (WT) mice were randomly assigned between the following four treatment groups equally: WT subarachnoid hemorrhage (SAH) sham + intraperitoneal (IP) normal saline (NS) + intracerebroventricular (ICV) NS (SAH sham + vehicle), WT SAH + IP NS + ICV NS (SAH + vehicle), WT SAH + IP deferoxamine (DFX) + ICV NS (SAH + IP DFX), and WT SAH + IP NS + ICV DFX (SAH + ICV DFX). Lab personnel performing surgical procedures were not the same as those performing cognitive assays to allow for appropriate blinding.

### SAH

The method used to induce SAH has been previously tested and validated in a mouse model [18]. After the mice were anesthetized with xylazine (10 mg/kg) and ketamine (12 mg/kg), SAH was performed as previously described by our lab using a standard stereotaxic instrument set-up (KOPF Instruments, Tujunga, CA, USA) [17]. To open the skin overlying the anterior skull, a midline incision was performed. Then, a burr hole was drilled into the anterior skull, 4.5 mm anterior to the bregma. Sixty microliters of autologous blood from a C57BL/6 wild-type blood donor mouse was injected over a 10-s period with a 27-gauge needle at a 40° caudal angle into the drilled burr hole. The needle was left in place for 5 min to prevent backflow of blood.

### Intracerebroventricular injection of deferoxamine

After the mice were anesthetized, intracerebroventricular injection was performed using a standard stereotaxic instrument set-up (KOPF Instruments, Tujunga, CA, USA). One burr hole was drilled 0.22 mm posterior to the bregma, 1 mm lateral, and 2.25 mm in depth to enter the ventricle. On SAH POD1, 24 h after the induced SAH, a single, non-repeated injection of 8 mg/kg of DFX was administered using pre-measured capillaries. Dosing was chosen based on dose-tolerance data generated by our lab (Table 1).

**Table 1:** Intracerebroventricular deferoxamine dose-tolerance chart

ICV dose	Total mice tested	Tolerance
0.8 mg/kg (~0.02 mg per mouse)	3	Well tolerated
8 mg/kg^a^ (~0.2 mg per mouse)	3	Well tolerated
80 mg/kg (~2 mg per mouse)	2	Immediately died

Wild-type mice were injected with a one-time intracerebroventricular (ICV) dose of deferoxamine diluted in normal saline, to test how well each dose would be tolerated

^a^The highest dose tolerated was chosen for all experimental procedures

### Intraperitoneal injection of deferoxamine

Starting on SAH POD1, 24-h after the induced SAH, the mice were given systemic injections of 200 mg/kg of DFX every morning, 30 min prior to cognitive test, until euthanization on SAH POD7. Dosage was chosen based on a previous publication testing systemic deferoxamine treatment in another mouse model of hemorrhagic stroke [19].

### TUNEL

All in vivo imaging was taken on SAH sham or SAH post-operative day (POD) 7 due to our lab’s previous publication showing SAH POD7 to have the most significant hippocampal cell damage [10]. Brain sections and HT-22 cells were stained with terminal deoxynucleotidyl transferase dUTP nick end labeling (TUNEL; Roche Life Science, Indianapolis, IN, USA). Slides were covered using Vectashield mounting medium with DAPI (Vector Laboratories, Burlingame, CA, USA) for nuclear counterstaining. HT-22 cells were counterstained with Hoechst 33258 (Sigma-Aldrich, Natick, MA, USA). Lab personnel interpreting TUNEL stains were not aware of the groups to which they were assigned.

### H&E

Coronal brain sections were stained with hematoxylin and eosin (H&E) (Poly Scientific R&D Corp., Bay Shore, NY, USA). The middle cerebral artery (MCA) lumen radius/wall thickness ratio was quantified using ImageJ software (NIH). Lab personnel interpreting H&E stains were not aware of the groups to which they were assigned.

### Confocal imaging

Eight-micrometer coronal brain sections from each experimental group were post-fixed and permeabilized, followed by being blocked with 10 % donkey serum. The sections were then stained with the following primary antibodies: goat anti-Iba1 (1:500) and rabbit anti-HO-1 (1:500) to identify HO-1 expression and HO-1 co-localization to microglia. The sections were then stained with the following secondary antibodies: donkey anti-goat 488 (1:250) and donkey anti-rabbit 594 (1:250). The sections were then counterstained with nuclei marker DAPI and sealed. The sections were viewed and images were taken on a Zeiss confocal microscope. Layers of images from the most anterior to the most posterior portion were taken, and a Z-stack image was created. ImageJ software was used for co-localization cell counting as well as co-localization heat maps. Co-localization percentage per high-powered field for each experimental group’s confocal Z-stacked images was obtained by dividing the total number of yellow-positive by the total number of DAPI-positive cells. Co-localization histograms were generated using the coloc-2 feature of ImageJ software. In brief, the program combines the green HO-1 single channel-1 saturation pixels with the red Iba-1/microglia single channel-2 saturation pixels to calculate the level at which they overlap. The *x*-axis represents channel-1 pixel intensity, while the *y*-axis represents channel-2 pixel intensity. Blue represents the lowest population frequency possible while yellow represents the highest. The lower the slope for each heat map, the more HO-1 staining there is per Iba-1/microglia staining.

### ELISA

Whole brain lysates were equally loaded onto a 96-well plate and cerebral IL-6 concentration was determined using ELISA Max Biolegend kit per manufacturer’s instructions. Media from microglia-neuron trans-well experiments were equally loaded onto a 96-well plate and culture TNF-α concentration was determined using ELISA Max Biolegend kit per manufacturer’s instructions (Biolegend, San Diego, CA, USA).

### Flow cytometry

All flow cytometry acquisition was performed on a FACSCalibur (BD Biosciences, San Jose, CA, USA), and analysis was completed using FlowJo software (FlowJo, LLC, Ashland, OR, USA). Cells were isolated from whole brain or blood and re-suspended in FACS buffer (1 % bovine albumin, 2 mM ethylenediaminetetraacetic acid (EDTA), and 0.05 % NaN_3_ in phosphate-buffered saline (PBS)). To block unspecific sites, the cells were first stained with CD16/32 Trustain (1:100; Biolegend, San Diego, CA, USA). The cells were then washed with FACS buffer and stained with the following fluorescent-tagged antibodies: PE-Gr-1 and PeCy7-CD11b (1:100; Biolegend, San Diego, CA, USA). To identify the myeloid cell populations in the blood and brain, CD45^+^ cells were gated off of a CD45/SSC-H dot plot. Then, using a Gr-1/CD11b dot plot, macrophages were identified as CD11b^hi^/Gr-1^lo^ while neutrophils were CD11b^hi^Gr-1^hi^ (Additional file 1: Figure S1). To measure the total mitochondrial superoxide anion production, whole brain cell lysates were incubated with MitoSOX red mitochondrial superoxide indicator per manufacturer’s instructions (5 μM; Life Technologies/ThermoFisher, Cambridge, MA, USA) and cells positive in the FL-2 channel were reported. Appropriate unstained controls for each channel were used to determine stained cell populations.

### Primary microglia and neuronal HT-22 cells—trans-wells

Microglia cells were isolated from the brains of neonatal mice using the Neural Tissue Dissociation Kit (P) (Miltenyi Biotec, Cambridge, MA, USA). The resulting mixed glia culture, containing astrocytes and microglia, was cultivated in media containing macrophage-colony stimulating factor (M-CSF). After 1 week of cultivation, the microglia were collected and grown on 3-μm cell culture inserts (EMD Millipore, Billerica, MA, USA). Murine hippocampal neuronal HT-22 cells were grown on six-well plates in normal media without M-CSF. For trans-well assays, the inserts with microglia were placed on top of the HT-22 neuron wells; microglia and HT-22 neuronal cells shared media during experiments. A total of 200 μl of whole blood was collected from the submandibular vein of a wild-type donor mouse and placed into 10 ml of PBS to keep the blood from clotting. The whole blood in PBS was then centrifuged at 2000 rpm for 5 min. The plasma, leukocyte, and platelet layers were aspirated, leaving an erythrocyte pellet. The erythrocytes were washed in an additional 10 ml of PBS and centrifuged at 2000 rpm for 5 min. The supernatant was aspirated and the erythrocyte pellet was re-suspended in 2 ml of PBS; 100 μl of this RBC suspension in PBS was added to the top microglia-containing chamber of the microglia-neuron trans-well; after 1-h of blood exposure, DFX (100 μM) or vehicle was added to the microglia chamber, and an additional hour of incubation was completed.

### Western blot

Primary microglial cell lysates were equally loaded onto a polyacrylamide gel and transferred to an immune-blot PVDF membrane (Bio-Rad, Hercules, CA, USA). Membranes were blocked with 5 % milk and stained with rabbit HO-1 antibody (1:1000, Abcam, Cambridge, MA, USA) and mouse vinculin antibody (1:1000, Sigma-Aldrich, Natick, MA, USA).

### Morris water maze

Cognitive performance was assessed using the Morris water maze as previously described, with minor modifications made by our lab [17]. In brief, after 7 days of acquisition, SAH sham or SAH was performed. On the day of SAH sham or SAH surgical procedure, the mice were not tested on the Morris water maze. The mice resumed testing on the Morris water maze on POD1. The mice to be treated with DFX were then either given daily IP DFX injections starting on POD1 and ending on POD7 or a one-time ICV injection of DFX on POD1. Additionally, mice that received daily IP DFX injections also got a one-time ICV injection of NS on POD1, and the mice that received a one-time ICV DFX injection also got daily IP NS injections from POD1 to POD7. SAH sham or SAH mice that were not treated with DFX, each received daily IP NS from POD1 to POD7 and a one-time injection of ICV NS on POD1 (Table 2). Spatial memory testing, measured by time to reach goal platform and consisting of 1-trial in the morning per animal per day, was started on SAH POD1 and continued for 7-days. On SAH POD4, the goal platform was moved to the opposite side of the maze (spatial reversal), while visual cue locations were unchanged. An investigator blinded to treatment groups performed the maze procedures.

**Table 2:** Wild-type experimental mouse groups

Mouse group	Abbreviation	Explanation
WT SAH sham + IP NS + ICV NS	SAH sham + vehicle	Mice received subarachnoid hemorrhage (SAH), sham surgical procedure, daily intraperoteneal (IP) injections of normal saline (NS) starting on post-operative day (POD) 1 and ending on POD7, and a one-time intracerebroventricular (ICV) injection of NS on POD1.
WT SAH + IP NS + ICV NS	SAH + vehicle	Mice received SAH surgical procedure, daily IP injections of NS starting on POD1 and ending on POD7, and a one-time ICV injection of NS on POD1.
WT SAH + IP DFX + ICV NS	SAH + IP DFX	Mice received SAH surgical procedure, daily IP injections of deferoxamine (DFX) starting on POD1 and ending on POD7, and a one-time ICV injection of NS on POD1.
WT SAH + IP NS + ICV DFX	SAH + ICV DFX	Mice received SAH surgical procedure, daily IP injections of NS starting on POD1 and ending on POD7, and a one-time ICV injection of DFX on POD1.

Wild-type (WT) mice were randomly assigned between the four treatment groups listed in the table. Each mouse was exposed to all surgical procedures and injections as specified in the explanation. The abbreviation listed in the table was used throughout the results including figures and figure legends for simplicity.

### Statistical analysis

Multiple experimental groups were compared using repeated-measures two-way ANOVA with Bonferroni’s post hoc test for in vivo TUNEL staining and HO-1/Iba1 co-localization of multiple brain regions, and the results are presented as the mean ± SD (GraphPad Prism). Morris water maze data was presented as the mean ± SEM (GraphPad Prism) and analyzed using repeated-measures two-way ANOVA with Bonferroni’s post hoc test. For all other statistical comparisons, multiple experimental groups were compared using one-way ANOVA with Bonferroni’s post hoc test, and the results are presented as the mean ± SD (GraphPad Prism). Differences were considered significant at *P* < 0.05.

### Study approval

All procedures involving animals were approved by the IACUC and the Radiation Safety Office (RSO) of Beth Israel Deaconess Medical Center.

## Results

### Intracerebroventricular deferoxamine injection dose-tolerance in wild-type mice

In order to determine the dose of DFX to be injected into the intracerebroventricular (ICV) space, a dose-tolerance chart was generated using WT mice (Table 1). Since 8 mg/kg was the highest dose tested that was well tolerated by the mice, this dose was chosen for further experimental procedures.

### Degree of deferoxamine neuroprotection and cognitive improvement after SAH depends on drug administration route and is vasospasm-independent

**Fig. 1:**
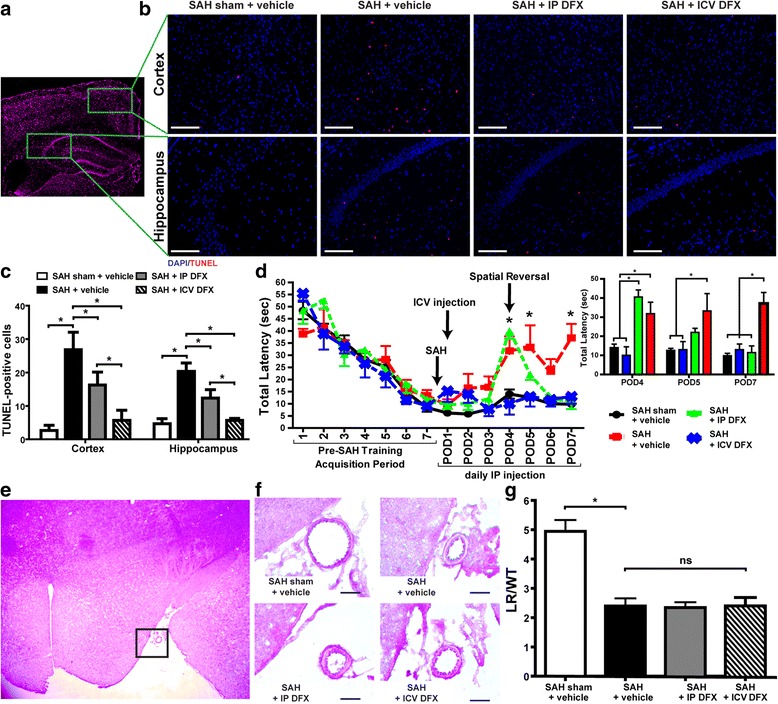
Deferoxamine provides vasospasm-independent neuroprotection and improves cognitive outcome after subarachnoid hemorrhage—cortical and hippocampal damage, cognitive outcome, and middle cerebral artery (MCA) vasospasm were measured in the following treatment groups in wild-type (WT) mice: subarachnoid hemorrhage (SAH) sham + vehicle, SAH + vehicle, SAH + intraperitoneal (IP) deferoxamine (DFX), and SAH + intracerebroventricular (ICV) DFX. **a** Image stained with DAPI (*magenta*) located at the anterior hippocampus. **b** Representative TUNEL (*red*) stained images of cortical and hippocampal sections from each treatment group with DAPI (*blue*) nuclei counterstain on post-operative day (POD) 7 (scale bar = 20 μm). **c** Quantification of TUNEL-positive cells for each group. The least amount of cortical and hippocampal damage was seen in the SAH + ICV DFX group followed by the SAH + IP DFX group. (Two-way ANOVA *P* < 0.05; *P < 0.05; *n* = 5 per group). **d** Morris water maze testing of WT mice cognitive outcome for each group. *Inset*, bar graph of data for POD4, 5, and 7 show the SAH + ICV DFX mice performed significantly better than the SAH + vehicle group on POD4 and 5 and significantly better than the SAH + IP DFX on POD4. Both SAH + IP DFX and SAH + ICV DFX groups performed significantly better than SAH + vehicle mice on POD7 (two-way ANOVA *P* < 0.05; *P < 0.05; *n* = 5 per group). **e** Image stained with hematoxylin and eosin (H&E) located adjacent to the anterior hippocampus. **f** Representative H&E stained images of MCA from each group on POD7 (scale bar = 10 μm). **g** Quantification of MCA vasospasm measured by lumen radius/wall thickness (LR/WT) quotient. DFX did not decrease vasospasm seen in the SAH + vehicle group (one-way ANOVA *P* < 0.05; *P < 0.05 SAH sham + vehicle versus SAH + vehicle; *n* = 5 per group)

All mice used had similar size and weight and appeared otherwise healthy prior to any surgical procedure. The WT mice were randomly assigned between the following four treatment groups: subarachnoid hemorrhage (SAH) sham + vehicle, SAH + vehicle, SAH + intraperoteneal (IP) DFX, and SAH + ICV DFX (Table 2). Neuronal damage and vasospasm were assessed for all of the treatment groups on POD7, based on our previous work [10]; cognitive outcome was assessed on POD1–7 using the Morris water maze (Fig. 1a–g). The SAH + vehicle mice had significantly increased cortical and hippocampal cellular damage compared to the SAH sham + vehicle mice, while the SAH + IP DFX mice and SAH + ICV DFX mice had markedly decreased cortical and hippocampal damage as compared to the SAH mice (ANOVA *P* < 0.05; *P* < 0.05 between groups; *n* = 5; Fig. 1b, c). The one-time ICV DFX injection decreased cortical and hippocampal damage to a greater extent than daily IP DFX injections (*P* < 0.05 versus SAH + IP DFX; Fig. 1b, c). Differences in cognitive outcome arose between the treatment groups on POD4, 5, and 7. The SAH + ICV DFX treatment group resulted in better cognitive performance than the SAH + vehicle and SAH + IP DFX groups on POD4 and the SAH + vehicle group on POD5. Additionally, both DFX treatment groups after SAH improved cognitive function when compared to the SAH + vehicle group on POD7 (ANOVA *P* < 0.05; *P* < 0.05 between groups; *n* = 5; Fig. 1d). Although the SAH + group had a significantly lower lumen radius to wall thickness (LR/WT) of the middle cerebral artery (MCA) than the SAH sham + vehicle group, DFX treatment had no effect on this SAH-induced MCA vasospasm (ANOVA *P* < 0.05; *P* < 0.05 SAH sham + vehicle versus SAH + vehicle; *n* = 5; Fig. 1f, g).

**Fig. 2:**
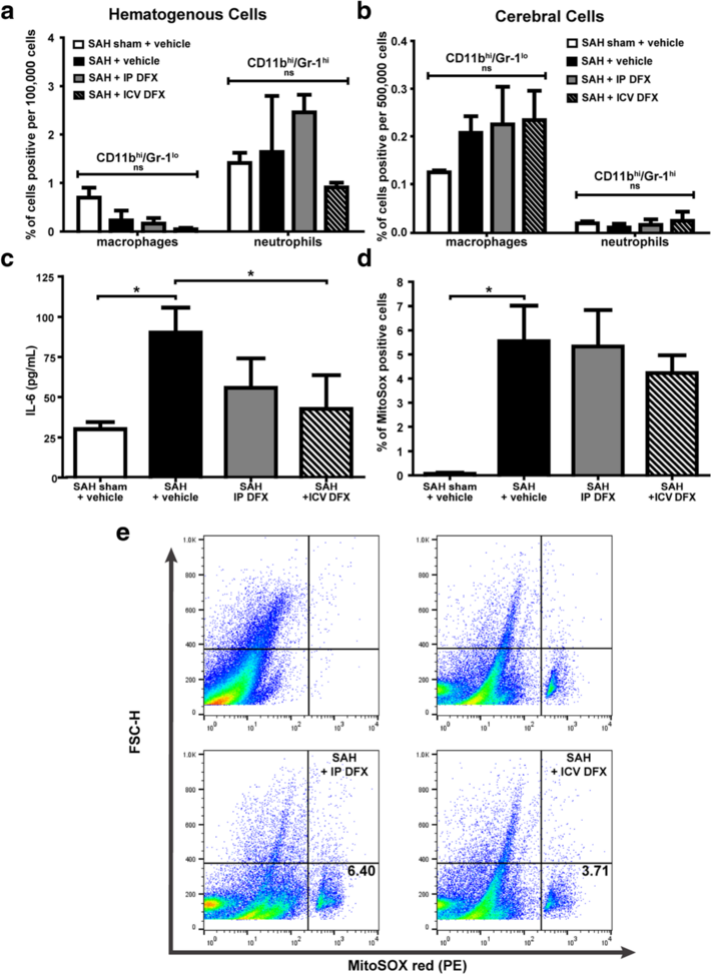
ICV DFX reduces cerebral inflammatory milieu after SAH—hematogenous and cerebral myeloid cell populations, cerebral IL-6 concentration, and cerebral mitochondrial superoxide anion were measured in the following treatment groups on POD7 in WT mice: SAH sham + vehicle, SAH + vehicle, SAH + IP DFX, and SAH + ICV DFX. **a** Flow cytometry of hematogenous cell populations show no significant change between any of the groups (one-way ANOVA; *n* = 4 per group). **b** Flow cytometry of cerebral myeloid cell populations show a trend towards increase in the microglia/macrophage populations of the SAH + vehicle group versus the SAH sham + vehicle group; but no significant changes between any of the other groups (one-way ANOVA; *n* = 4 per group). **c** Cerebral IL-6 concentration as measured by ELISA was significantly increased in the SAH + vehicle group (*P < 0.05 versus the SAH sham + vehicle group) and markedly decreased in the SAH + ICV DFX group (*P < 0.05 versus the SAH + vehicle group) (one-way ANOVA *P* < 0.05; *n* = 4 per group). **d** Cerebral mitochondrial superoxide anion (MitoSox) positive cells measured by flow cytometry did not show significant decrease in the SAH + ICV DFX group versus the SAH + vehicle group and the SAH + IP DFX group (one-way ANOVA *P* < 0.05; *n* = 4 per group). **e** Representative flow cytometry dot plots from each group. The Q3 percentage (shown) represents the percentage of cells positive for MitoSOX red

Next, we determined whether or not DFX had an effect on cerebral and hematogenous myeloid cell populations; it did not (*n* = 4; Fig. 2a, b). Subsequently, we measured the effects of DFX on cerebral inflammatory milieu after SAH on POD7 by quantifying interleukin (IL)-6 and mitochondrial superoxide anion in whole brain lysates. In the SAH + vehicle group, cerebral IL-6 production was increased as compared to control (ANOVA *P* < 0.05; *P* < 0.05 versus SAH sham + vehicle; *n* = 4; Fig. 2c); ICV DFX treatment markedly decreased the concentration of cerebral IL-6 after SAH while IP DFX did not (*P* < 0.05 versus SAH + vehicle; *n* = 4; Fig. 2c). Additionally, the SAH + vehicle group had increased mitochondrial superoxide anion production as compared to the control (ANOVA *P* < 0.05; *P* < 0.05 versus SAH sham + vehicle; *n* = 4; Fig. 2d, e), and while not statistically significant, there was a trend towards decreased mitochondrial superoxide anion in the SAH + ICV DFX treatment group as compared to the SAH + vehicle group (*n* = 4; Fig. 2d, e).

### Microglial HO-1 is sufficient for deferoxamine neuroprotection in an in vitro model

**Fig. 3:**
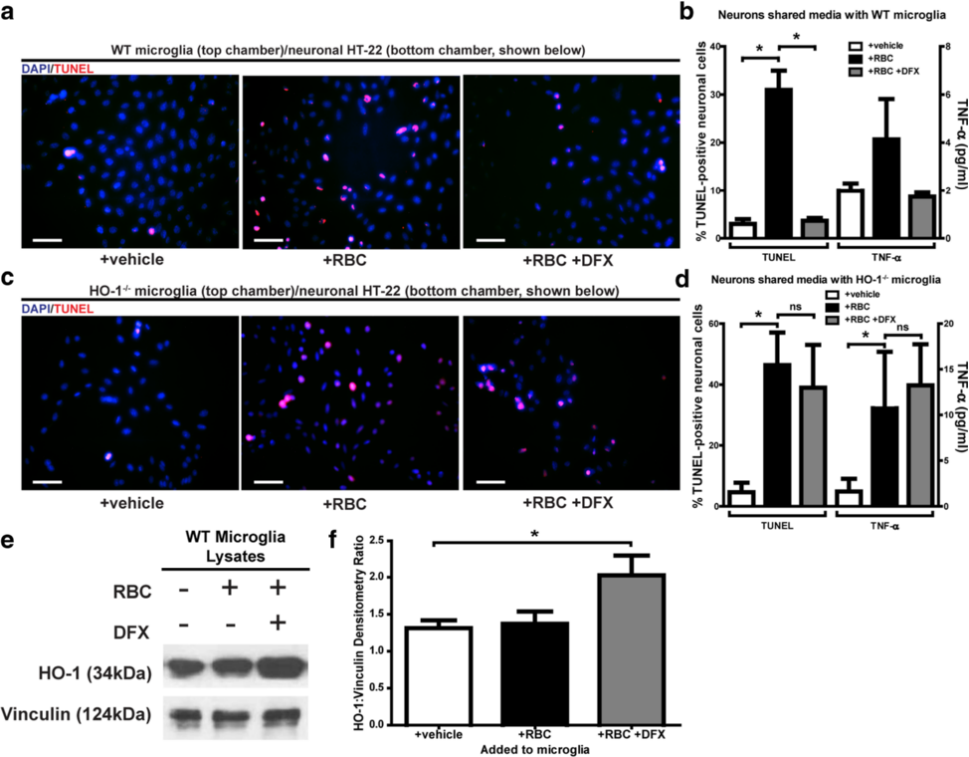
Microglial HO-1 has a role in deferoxamine protection from red blood cell (RBC)-induced neuronal damage—Trans-wells with WT or HO-1^−/−^ primary microglia were plated with hippocampal neurons (HT-22) and treated with vehicle, red blood cells (RBCs), or RBCs with DFX. **a** Representative TUNEL (*red*) stained images of neurons from each WT primary microglia trans-well with DAPI (*blue*) nuclei counterstain (all scale bars = 20 μm). **b** Quantification of TUNEL-positive neurons and TNF-α concentration from each WT primary microglia trans-well; RBC exposure significantly increased neuronal damage while DFX significantly reduced this RBC-induced damage (one-way ANOVA *P* < 0.05; *P < 0.05; *n* = 3 per group). Trend towards increased TNF-α production in the trans-well assays incubated with RBCs (one-way ANOVA *P* < 0.05; *n* = 3; **b**); trend towards decreased TNF-α production in the trans-well assays treated with DFX after RBC exposure (*n* = 3; **b**). **c** TUNEL stained images of neurons from each HO-1^−/−^ primary microglia trans-well with DAPI nuclei counterstain. **d** Quantification of TUNEL-positive neurons and TNF-α concentration from each HO-1^−/−^ primary microglia trans-well; RBC exposure significantly increased neuronal damage as well as TNF-α production, and DFX did not reduce this RBC-induced damage or TNF-α concentration (one-way ANOVA *P* < 0.05; *P < 0.05; *n* = 3 per group). **e** Western blot of primary microglial lysates from each group. **f** Quantification of bands from Western blot of primary microglial lysate; showed that HO-1 protein expression significantly increased in WT primary microglia culture exposed to RBCs with DFX (one-way ANOVA *P* < 0.05; *P < 0.05; *n* = 3 per group)

To determine if DFX treatment of microglia was sufficient to reduce neuronal damage, we performed in vitro trans-well assays with WT microglia and hippocampal neuronal HT-22 cells. WT microglia incubated with red blood cells (RBCs) for 2 h demonstrated marked neuronal damage by the trans-well assay (ANOVA *P* < 0.05; *P* < 0.05 versus control; *n* = 3; Fig. 3a, b). Additionally, there was a trend towards increased TNF-α production in the trans-well assays incubated with RBCs (ANOVA *P* < 0.05; *n* = 3; Fig. 3b). RBC-induced neuronal damage was significantly reduced when DFX was added 1 h after RBC exposure began, for a total RBC exposure of 2 h (*P* < 0.05 versus RBC only exposure; *n* = 3; Fig. 3a, b). Further, there was a trend towards decreased TNF-α production in the trans-well assays treated with DFX after RBC exposure (*n* = 3; Fig. 3b). To determine if the HO-1 pathway in microglia was involved, we repeated the trans-well experiment using HO-1^−/−^ microglia. In the trans-well assays with HO-1^−/−^ microglia, neuronal damage and TNF-α production was notably increased in trans-wells exposed to RBCs for 2 h (ANOVA *P* < 0.05; *P* < 0.05 versus control; *n* = 3; Fig. 3c, d). But unlike the WT microglia trans-wells, RBC-induced neuronal damage was *not* significantly reduced in neurons underlying HO-1^−/−^ microglia when DFX was added (*n* = 3; Fig. 3c, d). Further, TNF-α concentrations remained markedly elevated in assays with HO-1^−/−^ microglia after DFX addition to the RBC-treated trans-wells (*n* = 3; Fig. 3d).

Next, we sought to determine why the loss of microglial HO-1 diminished the neuroprotective effects of DFX. First, we measured the HO-1 protein expression in primary microglial lysates exposed to vehicle, RBCs, or RBCs with DFX. There was increased HO-1 protein expression in the microglia treated with RBCs and DFX as compared to vehicle (ANOVA *P* < 0.05; *P* < 0.05 versus control; *n* = 3; Fig. 3e, f).

### Intracerebroventricular deferoxamine increases in vivo microglia and HO-1 co-localization

**Fig. 4:**
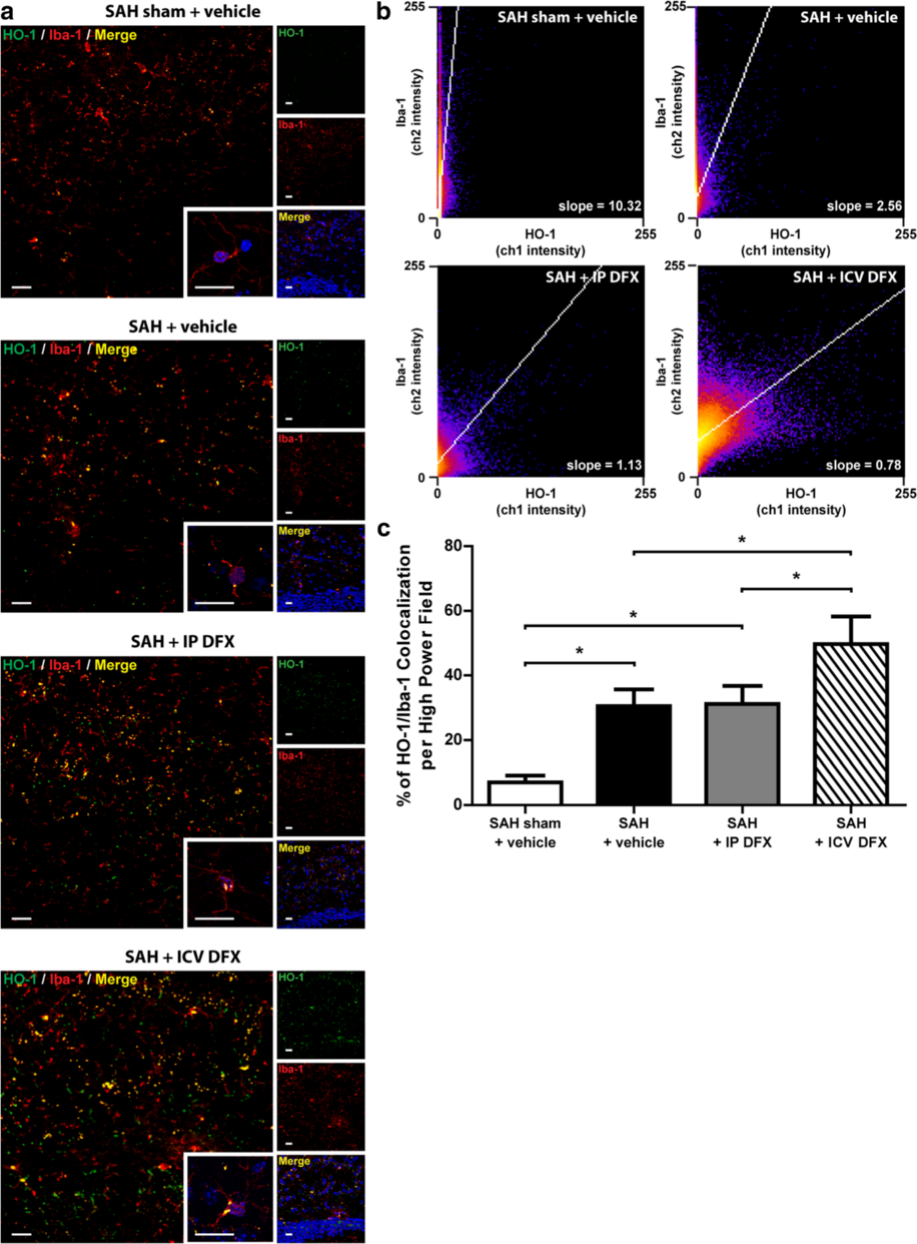
ICV DFX treatment after SAH causes an increase in HO-1/microglia co-localization—Confocal Z-stacked images were taken of the following treatment groups on POD7 in WT mice: SAH sham + vehicle, SAH + vehicle, SAH + IP DFX, and SAH + ICV DFX. **a** Representative confocal images of each experimental group. All images were taken adjacent to the anterior hippocampus, which can be visualized by the DAPI merged image at the bottom right tile. Each color represents the following: *green* = HO-1; *red* = Iba-1/microglia; *yellow* = overlap of green and red/co-localization of HO-1 and microglia; *blue* = DAPI nuclei counterstain. All *scale bars* = 20 μm. **b** Co-localization heat maps for each experimental group. The *x*-axis represents channel-1 (HO-1) pixel intensity, while the *y*-axis represents channel-2 (Iba-1) pixel intensity. *Blue* represents the lowest population frequency possible while *yellow* represents the highest. The lower the slope for each heat map, the more HO-1 staining there is per Iba-1/microglia staining. **c** The percent quantification of co-localization per high-powered field for each experimental group’s confocal Z-stacked images. Co-localization percentage was obtained by dividing the total number of yellow-positive cells by the total number of DAPI-positive cells. The highest HO-1/Iba-1 co-localization percentage was seen in the SAH + ICV DFX group, followed by the SAH + IP DFX and SAH + vehicle group (one-way ANOVA *P* < 0.05; *P < 0.05; *n* = 3 per group)

We wanted to validate the results we observed in our in vitro model, in vivo. That is, we wanted to examine whether ICV DFX increased microglial HO-1 expression in our in vivo SAH model. To see if DFX caused an increase in microglia and HO-1 co-localization, we took Z-stacked confocal images of brain sections adjacent to the anterior hippocampus, stained for microglia (Iba-1 positive) and HO-1 of all four of our in vivo experimental groups (Table 2). HO-1 co-localization to microglial cells on these in vivo sections from lowest to highest was as follows: SAH sham + vehicle, SAH + vehicle, SAH + IP DFX, and SAH + ICV DFX (ANOVA *P* < 0.05; *P* < 0.05 between groups; *n* = 3; Fig. 4a–c).

### Microglial/macrophage HO-1 is critical to intracerebroventricular deferoxamine neuroprotection and cognitive improvement after SAH

**Fig. 5:**
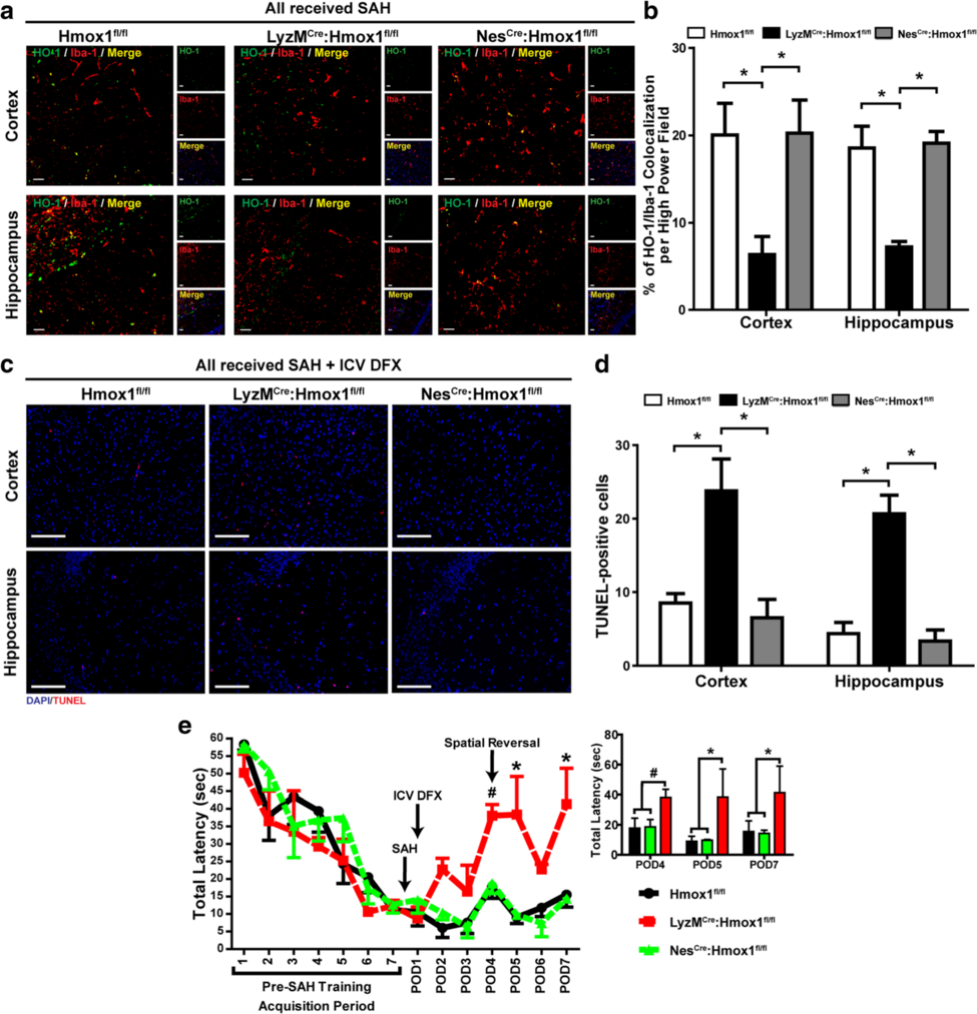
Macrophage/microglial HO-1 is critical to ICV DFX neuroprotection and improvement in cognitive outcome after SAH—Cortical and hippocampal damage as well as cognitive outcome were measured after SAH induction and ICV DFX treatment in the following HO-1 conditional knockouts: Hmox1^fl/fl^, LyzM^Cre^:Hmox1^fl/fl^, and Nes^Cre^:Hmox1^fl/fl^. **a** Representative confocal images of cortical and hippocampus sections for each genotype after SAH on POD7. Each color represents the following: *green* = HO-1; *red* = Iba-1/microglia; *yellow* = overlap of green and red/co-localization of HO-1 and microglia; *blue* = DAPI nuclei counterstain. All *scale bars* = 20 μm. **b** The percent quantification of co-localization per high-powered field for each genotype’s Z-stacked images. Co-localization percentage was obtained by dividing the total number of yellow-positive cells by the total number of DAPI-positive cells. LyzM^Cre^:Hmox1^fl/fl^ SAH mice showed the least HO-1/Iba-1 co-localization percentage in both the cortex and hippocampus as compared to Hmox1^fl/fl^ and Nes^Cre^:Hmox1^fl/fl^ SAH mice (two-way ANOVA *P* < 0.05; *P < 0.05; *n* = 4 per group). **c** TUNEL (*red*) stained images of cortical and hippocampal sections from each group with DAPI (*blue*) nuclei counterstain on POD7 (*scale bar* = 20 μm). **d** Quantification of TUNEL-positive cells from each treated genotype. Both Hmox1^fl/fl^ and Nes^Cre^:Hmox1^fl/fl^ SAH mice showed significantly less cortical and hippocampal damage than LyzM^Cre^:Hmox1^fl/fl^ SAH mice when treated with ICV DFX. (Two-way ANOVA *P* < 0.05; *P < 0.05 between groups for cortical damage and **P < 0.05 between groups for hippocampal damage; *n* = 4 per group). **e** Morris water maze testing of HO-1 conditional knockout cognition after SAH induction and ICV DFX treatment. *Inset*, bar graph of data for POD4, 5, and 7 shows both Hmox1^fl/fl^ and Nes^Cre^:Hmox1^fl/fl^ SAH mice performed significantly better than LyzM^Cre^:Hmox1^fl/fl^ SAH mice on POD5 and 7 when treated with ICV DFX, with a similar trend on POD4. (Two-way ANOVA *P* < 0.05;^#^ *P* = 0.07 between groups on POD4; *P < 0.05 between groups on POD5 and 7; *n* = 4 per group)

We performed SAH on *LyzM*^*Cre*^*:Hmox1*^*fl/fl*^ mice lacking microglial, neutrophil, and all other myeloid HO-1 as well as in *Nes*^*Cre*^*:Hmox1*^*fl/fl*^ mice lacking neuronal and astrocyte HO-1 and *Hmox1*^*fl/fl*^ control mice and measured the amount of HO-1 co-localization to microglia in each. *LyzM*^*Cre*^*:Hmox1*^*fl/fl*^ SAH mice showed the least HO-1/Iba-1 co-localization percentage in both the cortex and hippocampus as compared to *Hmox1*^*fl/fl*^ and *Nes*^*Cre*^*:Hmox1*^*fl/fl*^ SAH mice on POD7 (two-way ANOVA *P* < 0.05; *P* < 0.05 between groups; *n* = 4; Fig. 5a, b). We then performed SAH and subsequent ICV DFX treatment on *LyzM*^*Cre*^*:Hmox1*^*fl/fl*^, *Nes*^*Cre*^*:Hmox1*^*fl/fl*^, and *Hmox1*^*fl/fl*^ control mice. ICV DFX-treated *LyzM*^*Cre*^*:Hmox1*^*fl/fl*^ mice after SAH showed significant cortical and hippocampal damage on POD7 as compared to ICV DFX treated *Hmox1*^*fl/fl*^ SAH controls, while ICV DFX treated *Nes*^*Cre*^*:Hmox1*^*fl/fl*^ mice after SAH did *not* (ANOVA *P* < 0.05; *P* < 0.05 between groups; *n* = 4; Fig. 5c, d). Cognitive protection of ICV DFX after SAH was tested on *Hmox1*^*fl/fl*^, *LyzM*^*Cre*^*:Hmox1*^*fl/fl*^, and *Nes*^*Cre*^*:Hmox1*^*fl/fl*^ mice. The *LyzM*^*Cre*^*:Hmox1*^*fl/fl*^ mice performed markedly worse than the *Hmox1*^*fl/fl*^ control mice (ANOVA *P* < 0.05; *P* < 0.05 between groups for POD5 and 7; *n* = 4 Fig. 5e) while *Nes*^*Cre*^*:Hmox1*^*fl/fl*^ mice performed just as well as *Hmox1*^*fl/fl*^ control mice and significantly better than *LyzM*^*Cre*^*:Hmox1*^*fl/fl*^ mice (*P* < 0.05; *P* < 0.05 between groups for POD5 and 7; *n* = 4 Fig. 5e).

## Discussion

In a mouse model of SAH, we found that DFX exerted neuroprotective effects by non-canonical mechanisms. (1) DFX improved cognitive outcomes and reduced cerebral damage, independent of vasospasm. (2) ICV DFX was the most neuroprotective. (3) ICV DFX decreased neuroinflammatory markers. (4) Microglial HO-1 is sufficient for DFX neuroprotection in an in vitro model of blood-induced inflammation. (5) ICV DFX neuroprotection and cognitive improvement is dependent on microglial/macrophage HO-1.

The iron-chelating agent, DFX, has been tested for therapeutic use in many animal neurological disease models including Huntington’s disease [20], traumatic brain injury [21–23], cerebral ischemia [24–26], and hemorrhagic stroke among others. DFX has been extensively studied in animal models of intraventricular hemorrhage (IVH) and intracerebral hemorrhage (ICH). In IVH animal models, DFX reduced ventricular enlargement, brain damage, and markers of post-hemorrhagic chronic hydrocephalus [27–31]. In ICH animal models, DFX has been shown to reduce brain damage [32–37], decrease neuroinflammation [34, 38–40], and improve cognitive outcome [38, 41]. Further, DFX was shown to reduce DNA damage [37, 40], oxidative stress [32, 33, 38], neuronal hemoglobin expression [42], and autophagy markers [43], following ICH. Additionally, in a germinal matrix hemorrhage model of neonatal rats, DFX reduced brain damage, ventricular dilation, and improved cognitive outcome [44].

Although DFX treatment has also been studied in SAH animal models, experiments using DFX treatment specifically in a mouse model of SAH are lacking. In rat models of SAH, DFX has been shown to decrease overall mortality, edema, oxidative stress, and neuronal death [15, 16]. Additionally, DFX treatment after SAH has been shown to decrease cortical apoptotic markers [16] and reduce markers of brainstem damage in rats [45], as well as reduce lipid peroxidation markers and improve sodium-potassium ATPase activity in guinea pigs [46]. Our study looked to further elucidate the mechanisms of DFX-induced neuroprotection in a mouse model of SAH.

In our current study, administration of DFX *after* induction of SAH in our mouse model was effective in reducing the cerebral inflammatory response. DFX administered via two different routes, reduced cortical and hippocampal damage after SAH on POD7; the greatest reduction was seen with ICV DFX, followed by IP DFX (Fig. 1b, c). Further, both IP and ICV DFX treatment improved cognitive outcome during a later phase of SAH POD7, but only ICV DFX treated mice showed early improvement on POD4 and 5. Although DFX administration effectively reduced brain damage (Fig. 1b, c) and improved cognitive outcome (Fig. 1d) after SAH, it had no effect on vasospasm (Fig. 1f, g). These results were interesting for two reasons. First, the IP dose of DFX is 25-fold greater than the ICV dose, and yet the ICV dose showed more neuroprotection and earlier cognitive improvement. A potential explanation is that ICV administration allows for proximity to the heme burden, while IP-administered DFX has to effectively cross the blood brain barrier. Second, DFX provided cerebral protection and improved cognition after SAH independent of any effect on vasospasm, similar to previous studies that showed DFX treatment had no effect on vascular response after SAH [47, 48]. Vasospasm-independent cerebral protection provided by DFX is not surprising when one considers recent clinical trials that effectively treated vasospasm but did not improve morbidity or mortality after SAH [7, 8].

DFX has been previously shown to have anti-inflammatory effects in hemorrhagic stroke [14]. We investigated whether these anti-inflammatory effects might be partly mediated by changes in immune cell populations, cerebral IL-6 concentration, or mitochondrial superoxide anion production. The SAH + vehicle group showed an upward trend in the cerebral microglial/macrophage cell population as compared to the SAH sham + vehicle group, but DFX did not significantly reduce this increase. Cerebral neutrophils and hematogenous populations of macrophages and neutrophils remained unchanged with DFX administration (Fig. 2a, b). Further, SAH caused a significant increase in both cerebral IL-6 concentrations and mitochondrial superoxide production as compared to sham; in the ICV DFX-treated group, cerebral IL-6 concentrations were reduced and a trend towards decreased reduction in mitochondrial superoxide production was present (Fig. 2c–e). These results indicate that ICV DFX may partially exert protective effects not by changing the total number of microglia/macrophage cells, but instead via modulation of the pro-inflammatory mechanics of these cells, while systemic DFX injection does not.

We investigated whether DFX neuroprotection was dependent on microglia using a microglia-neuron trans-well assay. These assays revealed that DFX offered protection against red blood cell (RBC) induced neuronal damage, even when DFX was added to microglia *after* RBC exposure had already begun (Fig. 3a, b). When we repeated these trans-well assays with HO-1^−/−^ microglia, DFX offered no neuroprotection (Fig. 3c, d). Additionally, we found that DFX treatment increased the protein expression of HO-1 in primary microglial culture (Fig. 3e, f). These results suggest that microglial HO-1 is critical to the mechanism of DFX neuroprotection, possibly, in part, by facilitating the increased expression of microglial HO-1. Our lab has previously shown that administration of carbon monoxide (CO) rescues the neuronal injury seen in co-cultures with HO-1^−/−^ microglia [17]. This indicates that the neuroprotective product of heme breakdown via microglial HO-1 in the context of microglia-neuron co-cultures is CO. Since this current work shows microglial HO-1 to be increased following DFX treatment, it is likely that increased CO production may be involved. Additionally, DFX would chelate the excess iron from heme breakdown, potentially leading to synergistic benefits produced by DFX administration: increased CO protection and decreased iron toxicity.

When we looked at confocal images of all of our experimental groups, we found that SAH markedly increased the co-localization of microglia and HO-1 compared to sham. Further, we found that ICV DFX treatment affected a significant increase in HO-1 expression within microglia while IP DFX did not (Fig. 4a–c). Because these results demonstrated that ICV DFX most effectively increased microglial HO-1 expression in vivo, we next sought to ascertain the necessity of microglial HO-1 for ICV DFX neuroprotection. Mice lacking myeloid HO-1 (*LyzM*^*Cre*^*:Hmox1*^*fl/fl*^) and mice lacking neuronal and astrocyte HO-1 (*Nes*^*Cre*^*:Hmox1*^*fl/fl*^) were exposed to SAH and then treated with ICV DFX. Interestingly, ICV DFX treatment after SAH protected the *Nes*^*Cre*^*:Hmox1*^*fl/fl*^ mice similarly to the *Hmox1*^*fl/fl*^. Conversely, *LyzM*^*Cre*^*:Hmox1*^*fl/fl*^ mice showed significantly more neuronal damage and cognitive dysfunction compared to *Hmox1*^*fl/fl*^ mice (Fig. 5c–e). This, together with the in vitro data, supported the hypothesis that myeloid HO-1, but not astrocyte or neuronal HO-1, was critical for ICV DFX to reduce brain damage and improve cognition after SAH.

It is still not completely clear why the lack of myeloid HO-1 would suppress DFX neuroprotection in vivo, but we speculate that the DFX mediated increase in myeloid HO-1 cannot occur in the myeloid HO-1 knockout (*LyzM*^*Cre*^*:Hmox1*^*fl/fl*^*)* mice. Without the increased myeloid HO-1 expression, the subsequent increase in neuroprotective CO production would be absent. In our previous work, we showed that the neuronal damage and cognitive dysfunction seen in *LyzM*^*Cre*^*:Hmox1*^*fl/fl*^ mice could be saved by administering external CO, showing that CO was the neuroprotective byproduct of myeloid HO-1 heme breakdown [17]. Since our current in vitro data suggests that microglial HO-1 is crucial for DFX neuroprotection, it is possible that increased myeloid HO-1 expression in vivo, due to DFX administration, could cause increased CO production and thus result in better protection. Further experimentation would be necessary to test this theory.

We chose the anterior circulation model [18] over the endovascular perforation model and acknowledge that there are limitations; however, we felt that the strengths of the anterior circulation model outweighed the weaknesses. In the anterior circulation model, the increase in intracranial pressure is less severe. Additionally, blood entering the subarachnoid space of a mouse in this method would be that of a donor mouse. On the other hand, the endovascular perforation method better approximates the intracranial pressure crises that can occur in SAH patients.

However, the anterior circulation model has a number of advantages over the endovascular perforation method. First, the amount of blood (60 μl) injected into each mouse, and the resultant increased intracranial pressure is consistent between mice. Because of this, we feel that results obtained using the anterior circulation method are better reproduced. Further, the lower mortality seen with this model is helpful when performing experiments on conditional knockouts, as well as dual injection procedures required for SAH and intracerebroventricular DFX. We have used the anterior circulation method in our past research and believe it to be suitable for our current research interests as well.

In stroke patients, DFX reduced serum markers of oxidative stress and increased antioxidant species [49], while in ICH patients, phase-I testing revealed DFX to be safe and well tolerated [50]. Currently, promising clinical trials investigating the use of DFX for ICH are underway [51]. Our research provides a platform for linear translation of DFX treatment into the human SAH population. Our data show that intracerebroventricular DFX yields the greatest neuroprotection via a mechanism that is dependent on microglial HO-1 and possibly a protective microglial polarization. Given the fact that high-grade SAH patients will have an external ventriculostomy drain (EVD) placed at admission, a feasibility study for the use of intracerebroventricular DFX in these patients should be explored in the future. Furthermore, monitoring patient HO-1 expression during DFX treatment for hemorrhagic stroke may help clinicians identify patients that are more likely to respond to treatment.

## Conclusions

ICV DFX treatment provides superior neuroprotection in a mouse model of SAH. Our results indicate that the mechanisms by which DFX provides neuroprotection after SAH may involve microglial/macrophage HO-1 expression. Monitoring patient HO-1 expression during DFX treatment for hemorrhagic stroke may help clinicians identify patients that are more likely to respond to treatment.

## Additional file

Additional file 1: Figure S1. Flow cytometry gating method for identification of hematogenous and cerebral cells. (TIF 9440KB)

## Abbreviations

BIDMC: Beth Israel Deaconess Medical Center
CO: Carbon monoxide
DFX: Deferoxamine
EDTA: Ethylenediaminetetraacetic acid
EVD: External ventriculostomy drain
Fl: Flox
H&E: Hematoxylin and eosin
HO: Heme oxygenase
HO-1/Hmox1: Heme oxygenase-1
IACUC: Institutional Animal Care and Use Committee
ICH: Intracerebral hemorrhage
ICV: Intracerebroventricular
IL-6: Interleukin six
IP: Intraperitoneal
IVH: Intraventricular hemorrhage
LR/WT: Lumen radius/wall thickness
MCA: Middle cerebral artery
M-CSF: Macrophage-colony stimulating factor
NS: Normal saline
PBS: Phosphate-buffered saline
POD: Post-operative day
RBC: Red blood cell
SAH: Subarachnoid hemorrhage
TNF-α: Tumor necrosis factor-alpha
TUNEL: Terminal deoxynucleotidyl transferase dUTP nick end labeling
WT: Wild-type
