# Supplementary figures and images for: Heme oxygenase-1-mediated neuroprotection in subarachnoid hemorrhage via intracerebroventricular deferoxamine
Source: J Neuroinflammation. 2016 Sep 13;13(1):244. doi: 10.1186/s12974-016-0709-1 (PMC5020472; doi:10.1186/s12974-016-0709-1)

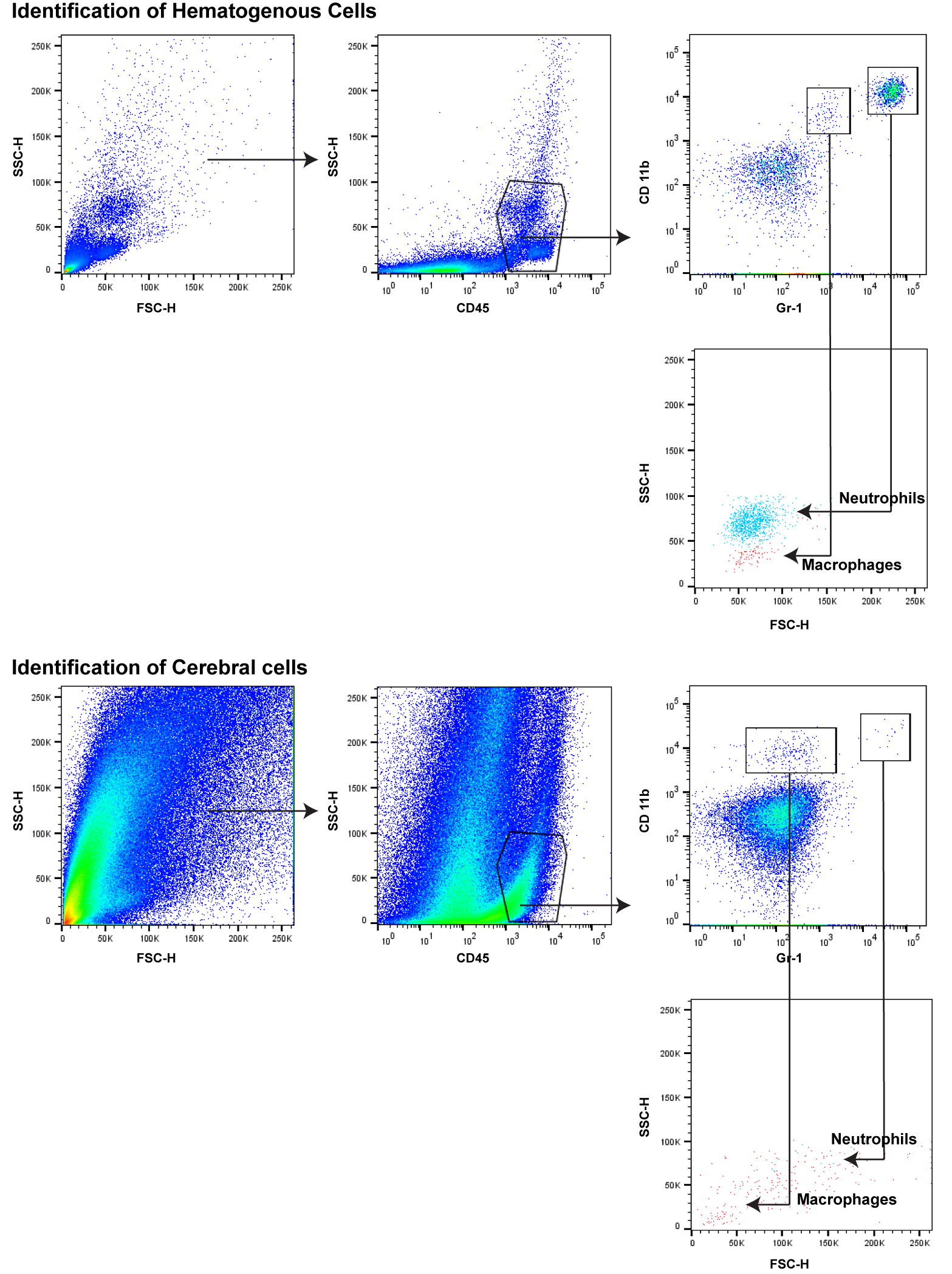

Supplement: Additional file 1: Figure S1. — Flow cytometry gating method for identification of hematogenous and cerebral cells. (TIF 9440KB) [file 12974_2016_709_MOESM1_ESM.tif]
